# Effects of walking on epigenetic age acceleration: a Mendelian randomization study

**DOI:** 10.1186/s13148-024-01707-w

**Published:** 2024-07-18

**Authors:** Guan-yi Chen, Chao Liu, Yu Xia, Ping-xiao Wang, Zi-yue Zhao, Ao-yu Li, Chu-qiao Zhou, Cheng Xiang, Jia-lin Zhang, Yi Zeng, Peng Gu, Hui Li

**Affiliations:** 1 Department of Orthopedics, The Second Xiangya Hospital of Central South University, Changsha, China; 2 Hunan Engineering Laboratory for Orthopedic Biomaterials, Changsha, China

**Keywords:** Aging, Walking, Epigenetic clock, Mendelian randomization study, Epigenetic age acceleration, Usual walking pace, GrimAge, Hannum, PhenoAge, Horvath

## Abstract

**Introduction:**

Walking stands as the most prevalent physical activity in the daily lives of individuals and is closely associated with physical functioning and the aging process. Nonetheless, the precise cause-and-effect connection between walking and aging remains unexplored. The epigenetic clock emerges as the most promising biological indicator of aging, capable of mirroring the biological age of the human body and facilitating an investigation into the association between walking and aging. Our primary objective is to investigate the causal impact of walking with epigenetic age acceleration (EAA).

**Methods:**

We conducted a two-sample two-way Mendelian randomization (MR) study to investigate the causal relationship between walking and EAA. Walking and Leisure sedentary behavior data were sourced from UK Biobank, while EAA data were gathered from a total of 28 cohorts. The MR analysis was carried out using several methods, including the inverse variance weighted (IVW), weighted median, MR-Egger, and robust adjusted profile score (RAPS). To ensure the robustness of our findings, we conducted sensitivity analyses, which involved the MR-Egger intercept test, Cochran’s *Q* test, and MR-PRESSO, to account for and mitigate potential pleiotropy.

**Results:**

The IVW MR results indicate a significant impact of usual walking pace on GrimAge (BETA = − 1.84, 95% CI (− 2.94, − 0.75)), PhenoAge (BETA = − 1.57, 95% CI (− 3.05, − 0.08)), Horvath (BETA = − 1.09 (− 2.14, − 0.04)), and Hannum (BETA = − 1.63, 95% CI (− 2.70, − 0.56)). Usual walking pace is significantly associated with a delay in epigenetic aging acceleration (EAA) (*P* < 0.05). Moreover, the direction of effect predicted by the gene remained consistent across RAPS outcomes and sensitivity MR analyses. There is a lack of robust causal relationships between other walking conditions, such as walking duration and walking frequency, on EAA (*P* > 0.05).

**Conclusion:**

Our evidence demonstrates that a higher usual walking pace is associated with a deceleration of the acceleration of all four classical epigenetic clocks acceleration.

**Supplementary Information:**

The online version contains supplementary material available at 10.1186/s13148-024-01707-w.

## Key messages

- In recent years, observational studies have found that there is a correlation between walking and aging, but the specific relationship is not clear
- We used a Mendelian randomization approach to test the causal effect of walking and sedentarism on epigenetic clock acceleration
- Mendelian randomization studies have found that faster walking speed may delay physiological aging through causal analysis at the genetic level

**Supplementary Information:**

The online version contains supplementary material available at 10.1186/s13148-024-01707-w.

## Introduction

The aging process is a complex phenomenon characterized by cumulative changes in life activities, resulting in diseases and eventual mortality [1]. While life expectancy has increased, the prevalence of chronic diseases remains high [2]. Traditional metrics like life span may not fully capture the intricacies of aging, prompting a contemporary shift in research focus toward interventions that address aging itself rather than isolated aspects like life span or individual diseases. Unlike chronological aging, biological aging is a modifiable process, and recent advancements in aging research have identified biomarkers, particularly methylation time clocks, as promising predictors of aging [3, 4]. In recent years, its plausibility as a predictor of biological age has been strongly supported [5–8]. DNA methylation, a crucial epigenetic process involving the addition of a methyl group to the cytosine ring, plays a pivotal role in growth, development, and aging. [9] It is a form of epigenetic modification in which DNA is directly methylated through covalent linkage of the methyl group to the fifth position of the cytosine ring to generate 5-methylcytosine (5mC) [10]. Dynamic changes in DNA methylation patterns, especially in CpG dinucleotide-rich regions, occur with age, leading to the development of epigenetic clocks that measure biological age [11]. Typically, CpGs in promoter regions are hypermethylated during aging, while other CpGs are hypomethylated [12]. DNA methylation patterns have been used as a measure of biological age, currently known as the epigenetic clock. These clocks, particularly those based on methylation, have demonstrated correlations with age-related morbidity, mortality, and various other factors. This underscores their potential to predict and identify risks associated with aging. As our understanding of aging advances, the focus on epigenetic measures provides insights into the intricate processes underlying aging, offering avenues for targeted interventions to promote healthier aging. This shift toward a more holistic view of aging, encompassing biological and epigenetic aspects, represents a promising approach in the quest for interventions to enhance the quality of life in aging populations.

The discovery and study of the epigenetic clock offers great opportunities to explore interventions in aging. Acceleration of aging, also known as epigenetic age acceleration (EAA), can be generated by comparing biological age with chronological age. Exploring the effects of different interventions on aging through changes in apparent age can provide means to delay and reverse aging.

The daily activity of walking is an essential daily activity for everyone. For individuals, walking is good for both physical and mental health [13, 14]. For society, walking can effectively reduce healthcare costs [15]. However, most of the previous studies were small-scale clinical intervention studies or observational studies, and the evidence had certain limitations, such as limited samples, lack of specificity, and lack of clear causality. In this study, we employed a Mendelian randomization (MR) approach to investigate the causal relationship between various walking conditions, including walking speed, walking duration, 4-week walking frequency, and epigenetic aging acceleration (EAA) at the genetic level. These walking conditions were compared against sedentary leisure behavior. Our findings reveal a causal relationship, indicating that faster walking is associated with slower epigenetic aging.

## Method

### Study design

In this study, a two-sample bidirectional Mendelian randomization (MR) approach was used to investigate the causal relationship between walking and epigenetic age acceleration (EAA). This MR method is typically employed to obtain the association between genetic variant exposure and genetic variant outcome in two different participant samples [16].

**Fig. 1:**
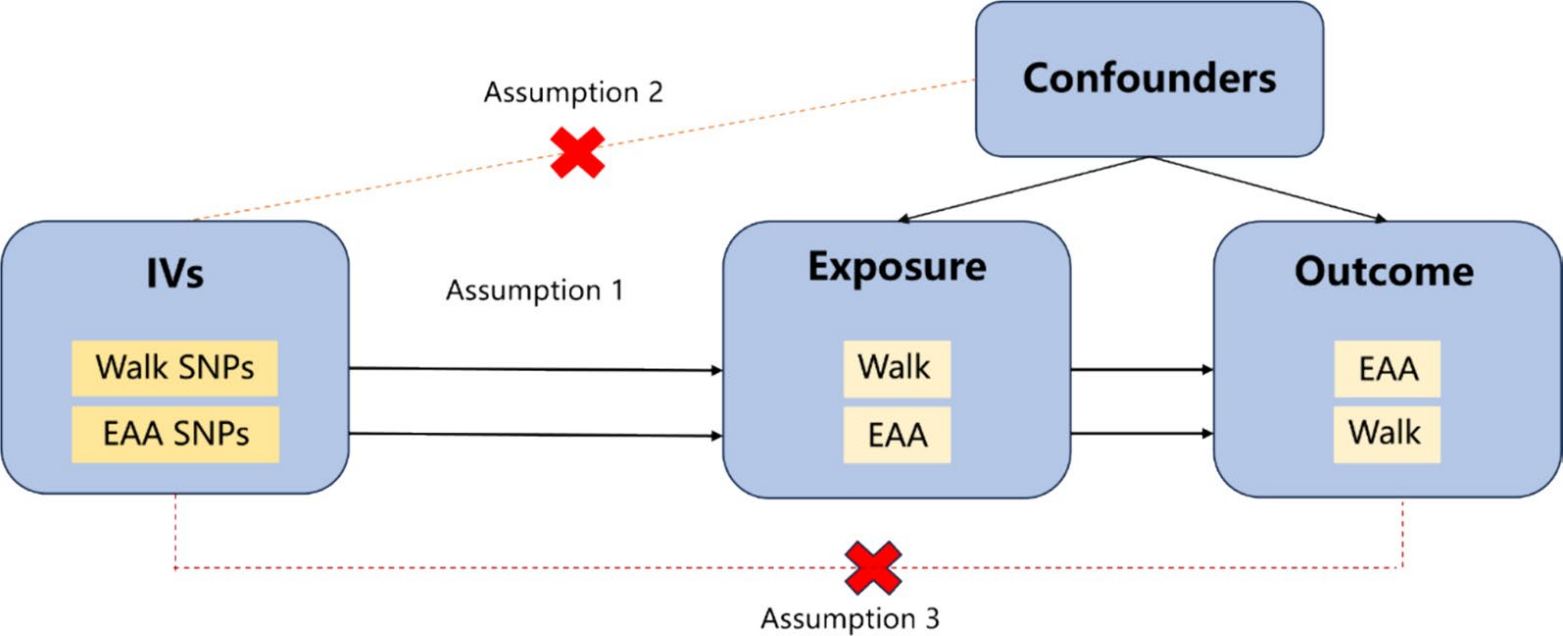
Schematic representation of the three assumptions and study design. *IVs* Instrumental variables. *SNPs* Single nucleotide polymorphisms. Assumption 1: The selected genetic IVs are robustly associated with the exposure. Assumption 2: The chosen IVs are not associated with potential confounders. Assumption 3: The IVs can only influence the risk of the outcome dependently through exposure

The MR study is based on three basic assumptions: (1) the instrumental variables (IVs) are strongly related to the exposure of interest; (2) the IVs are not affected by confounding factors; (3) the IVs represent the outcome caused by exposure rather than directly affecting the outcome (Fig. 1).

For hypothesis 1, SNPs that were strongly associated with exposure were selected as instrumental variables. For hypothesis 2, instrumental variables required the exclusion of confounders related to exposure and outcome. This criterion does not apply here due to the lack of intermediate confounders between aging, walking, and sitting. For hypothesis 3, we excluded instrumental variables that were strongly correlated with the outcome but weakly correlated with exposure, ensuring that the instrumental variables related to exposure were indeed causing the outcome.

We first explored the effects of walking and sedentary behavior (exposure) on epigenetic age acceleration (outcome) and then reversed the analysis to examine the effects of epigenetic age acceleration on walking and sedentary behavior.

### Data sources

Walking data were derived from the UK Biobank, a study of 501,726 UK residents aged between 40 and 69 years at the time of recruitment. Participants attended the assessment visits at 23 research centers in England, where a wide range of phenotypic data were collected. Participants provided informed consent, and ethical approval for the UK Biobank study was obtained from the Northwest National Research Ethics Committee (REC reference 11/NW/0274).

Genotyping, imputation, and quality control were performed by the UK Biobank. Genotyping was carried out using the UK BiLEVE Axiom array and the UK Biobank Axiom array, and imputation was done using the Haplotype Reference Consortium panel, which includes approximately 96 million variants. General walking speed data involved 459,915 individuals. Subjects were asked, “How would you describe your usual walking pace?” with options: slow pace, steady average pace, and brisk pace. Slow pace was defined as less than 3 miles per hour, steady as 3–4 miles per hour, and brisk as more than 4 miles per hour, excluding participants unable to walk.

Walking duration data involved 395,831 individuals, who were asked how many minutes they usually walked in a day. The average was taken if the duration varied greatly during the week. Data on the frequency of walking for pleasure in the last 4 weeks involved 328,320 individuals, who were asked, “How many times in the last 4 weeks did you go walking for pleasure?” Data were accessed from GWAS MRC IEU and further information can be found at UK Biobank [17]. Sedentary data involved 437,887 individuals, and candidate genetic tools for leisure sedentary behavior phenotypes were extracted from the IEU GWAS database [18].

Methylation clock data were derived from a meta-analysis of 28 cohorts of individuals of European ancestry, including 34,710 participants. The age of the sample in the meta-analysis ranged from 27.2 to 79.1 years (mean 54.8 years), with 57.3% being women. Epigenetic age was calculated using Horvath’s epigenetic age calculator software (DNA methylation age or by using scripts provided by Steve Horvath and Ake Lu). The measures used were age-adjusted Hannum age [19], the Intrinsic DNA methylation Horvath [20], PhenoAge [5], and GrimAge age [21]. Evaluate the following output: inner epigenetic age—“IEAA” faster, Hannum age—“AgeAccelerationResidualHannum” faster, phenotypic age—“AgeAccelPheno” faster, and GrimAge acceleration—“AgeAccelGrim.”

For each cohort, methylation values beyond ± 5 standard deviations were considered outliers and excluded from the analysis. For more comprehensive details regarding the samples and methodology, we refer readers to the original GWAS meta-study [22].

### Instrumental variables

In our study, we carefully selected independent single nucleotide polymorphisms (SNPs) that exhibited significant associations with the exposure at the genome-wide level (*p* < 5 × 10^− 8) to serve as instrumental variables (IVs). To prevent issues related to linkage disequilibrium, we excluded SNPs with an *r*^2 greater than 0.001 within a 10,000-kilobase (10,000 KB) range. Linkage disequilibrium is measured by two parameters: *r*^2 and KB. An *r*^2 value of 1 indicates complete linkage disequilibrium, while an *r*^2 value of 0 indicates complete random distribution. The KB parameter indicates the genomic region length considered for linkage disequilibrium, where closer genetic loci suggest a stronger genetic relationship [23].

We also tested the strength of genetic SNPs using the *F*-statistic. Traditionally, an *F*-statistic less than 10 indicates a weak instrumental variable, reflecting low power for the SNP-exposure association and potential bias. A weak instrumental variable explains the exposure with low genetic variation, meaning the strength of this association is not very high, thus differing from an invalid instrumental variable. The primary cause of weak instrumental variable bias is insufficient sample size.

The *F*-statistic is calculated as follows:

$$F = \left( {N - k - 1} \right)/k \, * \, R^{2} /\left( {1 - R^{2} } \right)$$

where *N* represents the sample size in the GWAS study, *k* represents the number of IVs, and *r*^2 is the coefficient of determination for the IV-exposure association.

### MR analysis

We applied various Mendelian randomization (MR) statistical methods, including inverse variance weighting (IVW), weighted median, weighted mode, and MR-Egger. Among these methods, IVW is considered the most crucial. IVW is proposed by Burgess and utilized in MR studies of multi-instrumental variables [24, 25]. IVW is generally acknowledged as the most accurate and stable method for estimating causality [24]. Assume that G {G1, G2,… GJ} represents the instrumental variables, X is the exposure factor, and Y is the outcome variable. For the instrumental variable G1, the effects on the exposure factor and the outcome are *β*_XG1_ and *β*_YG1_, respectively, with corresponding standard errors σ_βXG1_ and *σ*_*β*YG1_. Fixed or random effects models were then used to obtain causal effect sizes between exposures and outcomes. However, IVW assumes that SNPs are not pleiotropic, which may introduce significant bias if pleiotropy is present.

To address this concern, we conducted a horizontal pleiotropy test using the MR-Egger intercept. While IVW forces the intercept of the linear regression to be 0, MR-Egger measures the average pleiotropic effect between instrumental variables through the intercept term [26]. Additionally, we utilized MR-pleiotropy residual and outlier (MR-PRESSO) to identify and remove SNPs with pleiotropic effects [27]. MR-PRESSO calculated the squared residuals between the IVW results before and after the removal of each SNP, which were summed as the total sum of squared residuals. A larger sum of squared residuals indicated more significant horizontal pleiotropy, and SNPs with larger squared residuals were considered potential outliers. If outliers were present, the IVW results were recalculated after removing these outliers.

We assessed SNP heterogeneity through Cochran’s Q statistic [28]. Based on the results, we employed fixed or random effects models (fixed-effect model for *p* > 0.05, random-effect model in cases of heterogeneity).

To handle bias and assess causality, including situations with substantial variation arising from weak SNPs, we used robust adjusted profile scores (RAPS) [29]. RAPS proposes a consistent and asymptotically normal estimator by adjusting the profile score and tackling idiosyncratic pleiotropy through robustifying the adjusted profile score.

All MR analyses were conducted using the “MungeSumstats,” “TwoSampleMR,” and “MR-PRESSO” R packages in R statistical software (version 4.2.2). Further research methods can be found at MRC IEU TwoSampleMR.

## Result

### A bidirectional two-sample MR analysis

**Fig. 2:**
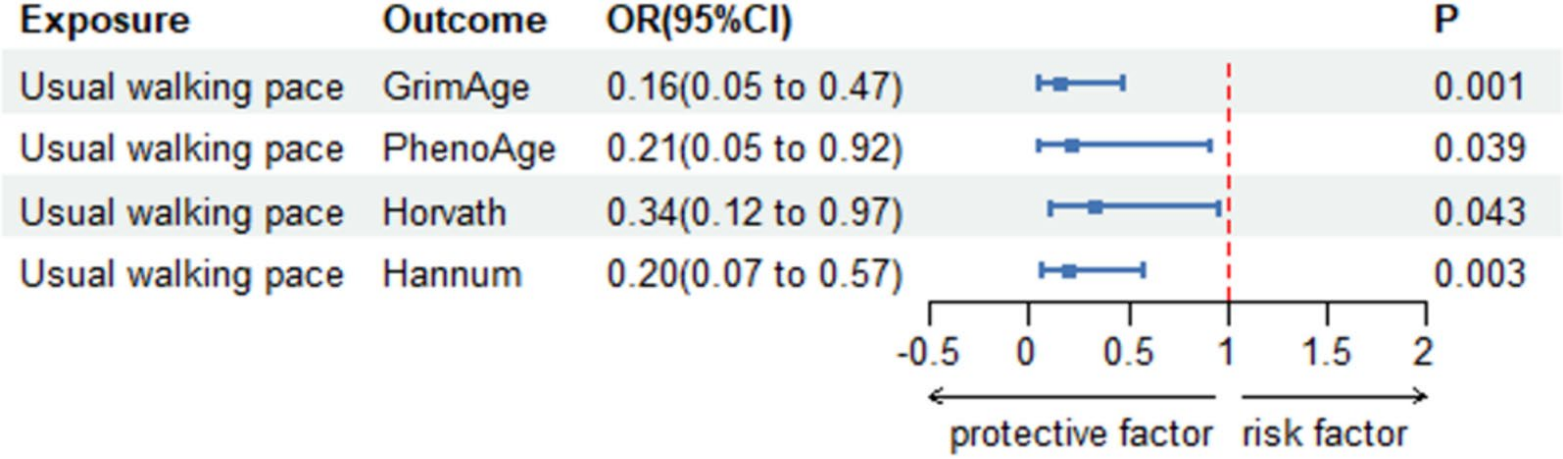
Causal estimates from genetically predicted usual walking speed to four epigenetic age acceleration (GrimAge, PhenoAge, Horvath, and Hannum) were obtained using IVW methods

**Fig. 3:**
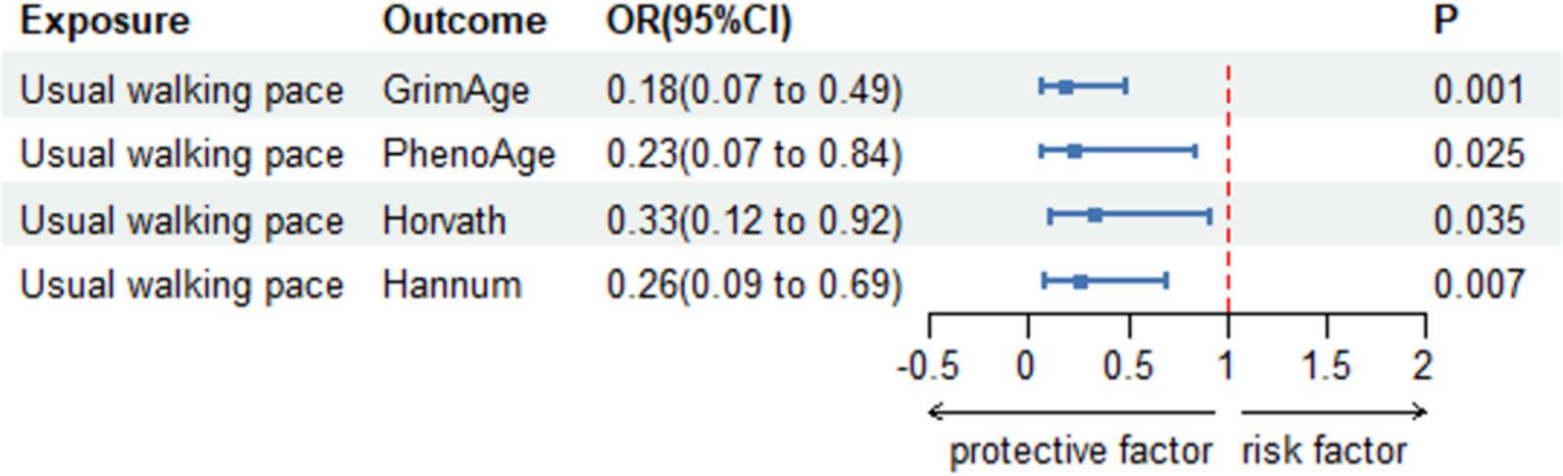
Causal estimates from genetically predicted usual walking speed to epigenetic clock acceleration (GrimAge, PhenoAge, Horvath, and Hannum) were obtained using RAP methods

We first calculated the *F*-statistics for the SNPs for usual walking speed, walking time, and walking frequency in the last four weeks, each of which had an *F*-statistic above 10. The detailed results of the *F*-statistics are provided in Supplementary Table 2. It can be considered that the selected SNPs are not weak instrumental variables. As shown in Table 1, after removal of linkage disequilibrium and deletion of repetitive SNPs, usual walking pace was inversely associated with the acceleration of the four classical aging clocks, GrimAge (-1.842(− 2.937, − 0.747), *P* < 0.001), PhenoAge (1.567 (3.052, 0.082), *P* = 0.039), Horvath (1.089 (2.142, 0.035), *P* = 0.043), and Hannum (− 1.626(− 2.695, − 0.557), *P* = 0.003) (Fig. 2). There is often a genetic causal relationship between walking speed and epigenetic age acceleration. The results obtained using RAPS method also proved this point, GrimAge (− 1.721(− 2.731, − 0.711) *P* < 0.001), PhenoAge (− 1.261(− 2.722, − 0.178), *P* = 0.025), Horvath (− 1.105079(− 2.131, − 0.079) *P* = 0.035), and Hannum (− 1.359455(− 2.353, − 0.365) *P* = 0.007) (Fig. 3). However, walking time and the frequency of walking in the last four weeks did not have a causal effect on the four epigenetic age accelerations (*P* > 0.05). We observed a significant positive association between leisure sedentary behavior and GrimAge EAA. However, there was a lack of reliable causality with the other three classical epigenetic clocks acceleration. (Table 1) We then performed inverse MR Tests, which showed no causal effects of acceleration of the four epigenetic clocks on walking speed, time, near-four-week frequency or leisure sedentary behavior (*P* > 0.05). (Supplementary Table 1).

**Table 1:** Primary Mendelian randomization estimates of walking and sedentarism on EAA

Exposures	Outcomes	IVW		Weighted median		MR-Egger		RAPS	
		Beta (95% CI)	*P*	Beta (95% CI)	*P*	Beta (95% CI)	*P*	Beta (95% CI)	*P*
Leisure sedentary behavior	GrimAge	0.780 (0.369,1.191)	< 0.001	1.100(0.488,1.711)	< 0.001	1.619(0− 0.337,3.575)	0.108	0.815(0.432,1.199)	< 0.001
	PhenoAge	0.387(− 0.137,0.912)	0.148	0.500(− 0.286,1.287)	0.213	1.963(− 0.533,4.459)	0.126	0.640(0.155,1.124)	0.010
	Horvath	0.114(− 0.329,0.556)	0.615	0.187(− 0.437,0.811)	0.557	0.090(− 2.020,2.200)	0.934	0.302(− 0.086,0.690)	0.127
	Hannum	− 0.137(− 0.598,0.323)	0.560	0.040(− 0.568,0.648)	0.896	1.532(− 0.640,3.703)	0.170	− 0.059(− 0.435, 0.317)	0.757
Usual walking pace	GrimAge	− 1.842(-2.937, − 0.747)	< 0.001	− 2.378(− 3.932, − 0.824)	0.003	− 2.256(− 6.979,2.467)	0.354	− 1.721(− 2.731, − 0.711)	< 0.001
	PhenoAge	− 1.567(− 3.052, − 0.082)	0.039	− 1.224(− 3.214, 0.766)	0.228	5.787(− 0.310,11.883)	0.069	− 1.261(− 2.722, − 0.178)	0.025
	Horvath	− 1.089(− 2.142, − 0.035)	0.043	− 0.777(− 2.270,0.715)	0.307	2.518(− 1.973,7.008)	0.277	− 1.105(− 2.131, − 0.079)	0.035
	Hannum	− 1.626(− 2.695,-0.557)	0.003	− 1.109(− 2.522,0.304)	0.124	− 1.336(− 5.931,3.259)	0.571	− 1.359(− 2.353, − 0.365)	0.007
	GrimAge	− 0.767(− 2.229,0.696)	0.304	− 0.129(− 1.946,1.688)	0.890	− 2.502(− 10.126,5.120) 0.536	− 0.719(− 2.010,0.573)	0.275	
Duration of walks	PhenoAge	0.066(− 2.271,2.403)	0.956	0.826(− 1.734,3.386)	0.527	− 9.326(− 20.011,1.360) 0.121	− 0.133(− 1.748,1.483)	0.872	
	Horvath	0.093(− 1.383,1.569)	0.901	0.848(− 0.932,2.628)	0.35	2.817(− 4.759,10.393)	0.49	− 0.150(− 1.456,1.155)	0.821
	Hannum	0.532(− 0.792,1.856)	0.431	0.445(− 1.317,2.207)	0.621	− 1.708(-8.352,4.936)	0.63	0.329(− 0.947,1.605)	0.613
Frequency of walking for pleasure in last 4 weeks	GrimAge	− 0.395(− 1.890,1.101)	0.605	− 0.375(0.637, − 1.932)	0.637	15.146(4.880,25.411)	0.03	− 0.244(–1.240,0.752)	0.631
	PhenoAge	− 0.619(− 1.933,0.696)	0.356	− 0.287(− 2.053,1.477)	0.749	1.700(− 11.238,14.638)	0.81	− 0.472(− 1.751,0.808)	0.470
	Horvath	0.333(− 0.722,1.388)	0.536	0.296(− 1.103,1.694)	0.679	2.266(− 8.150,12.677)	0.69	0.333(− 0.695,1.360)	0.526
	Hannum	0.224(− 0.803,1.251)	0.669	0.312(− 1.018,1.642)	0.646	6.531(− 3.621,16.683)	0.26	0.328(− 0.673,1.328)	0.521

*CI* Confident interval, *IVW* Inverse variance weighted, *RAPS* Robust Adjusted Profile Scores

### Stage II: sensitivity analysis

We calculated the statistical values of SNPs for the epigenetic age acceleration in the four methylation time clocks, and the *F* values were also all above 10. In the subsequent sensitivity analysis, the heterogeneity results revealed significant heterogeneity between walking time and PhenoAge EAA SNPs (*P* = 0.042), as well as between leisure-time sedentary behavior and Hannum EAA SNPs (*P* = 0.023). The newly calculated results using the random effects model indicated that there may still be no causal relationship between walking time and PhenoAge EAA (*P* = 0.956) or between leisure-time sedentary behavior and Hannum EAA (*P* = 0.559). All other results showed no heterogeneity (*P* > 0.05). Among the horizontal pleiotropy results, MR-Egger results showed that there may be horizontal pleiotropy between SNPs with accelerated PhenoAge and those with usual walking pace (*P* < 0.019). MR-PRESSO was employed to identify potential pleiotropic SNPs, revealing the presence of pleiotropic SNPs between leisure sedentary behavior and Hannum EAA (*P* = 0.030). However, the results remained unchanged even after the removal of outliers. The results of IVW method are reliable. There was no horizontal pleiotropy between the other SNPs for walking speed, time, frequency of walking in the last four weeks, or leisure sedentary behavior on the four epigenetic age accelerations (Table 2).

**Table 2:** Association walking with epigenetic age acceleration using heterogeneity test, pleiotropy test, MR-PRESSO and MR-Egger

Exposures	Outcomes	Cochran’s *Q* (*P*)	MR-Egger (*P*)	MR-PRESSO (*P*)
Leisure sedentary behavior	GrimAge	0.507	0.392	0.396
	PhenoAge	0.435	0.209	0.165
	Horvath	0.179	0.982	0.163
	Hannum	0.023	0.126	0.030
Usual walking pace	GrimAge	0.272	0.861	0.364
	PhenoAge	0.091	0.019	0.057
	Horvath	0.562	0.112	0.719
	Hannum	0.307	0.899	0.305
Duration of walks	GrimAge	0.299	0.660	0.458
	PhenoAge	0.042	0.113	0.080
	Horvath	0.303	0.490	0.424
	Hannum	0.878	0.517	0.856
Frequency of walking for pleasure in last 4 weeks	GrimAge	0.052	0.031	0.072
	PhenoAge	0.587	0.738	0.702
	Horvath	0.531	0.730	0.688
	Hannum	0.624	0.276	0.710

*MR-PRESSO* Pleiotropy residual and outlier, *RAPS* Robust adjusted profile scores

## Discussion

This represents the inaugural large-scale two-sample Mendelian randomization (MR) study uncovering the causal link between walking and epigenetic aging. Our results illuminate a consistent and significant causal association, indicating that increased walking speed correlates with the deceleration of epigenetic aging. Essentially, brisk walking appears to exert a beneficial influence on slowing down the aging process. Notably, this causal relationship persists uniformly across all four classical epigenetic clocks. Furthermore, a thorough sensitivity analysis was conducted, underscoring the robustness of our findings. The results maintained stability even after rigorous testing for horizontal pleiotropy and adjustments for heterogeneity. In contrast, alternate facets of walking, including walking duration and frequency over the past four weeks, did not exhibit resilient causality concerning accelerated epigenetic aging. Additionally, a comparative analysis using sedentary behavior revealed that leisurely sedentary behavior induced GrimAge EAA. While no conclusive causal link was identified in the analysis of sedentary behavior on the remaining three epigenetic clocks, the heightened correlation of GrimAge with behavioral lifestyle suggests a potential association between sedentary behavior and accelerated aging.

MR results offer valuable insights into the precise relationship between walking and the aging process. In previous observational studies, walking has consistently been recognized as closely linked to aging, with walking speed serving as a significant indicator of the aging process. As individuals age, their walking speed tends to slow down significantly, emphasizing the role of walking in assessing age-related changes [30, 31]. Simultaneously, several studies have identified a strong correlation between gait speed and the onset of various age-related physical conditions and adverse events. For instance, a Chinese study involving 3,009 individuals with an average age of 66.4 years found that slower walking speed was associated with a more pronounced future cognitive decline. This underscores the significance of gait speed as a comprehensive marker for assessing not only the aging process, but also a range of related health outcomes and cognitive changes. [32, 33] Indeed, one study has already used walking speed to train a new epigenetic clock to evaluate physical condition. [34]

These additional studies have consistently demonstrated that increased gait speed is associated with a reduced likelihood of cognitive impairment and enhanced cardiovascular and cerebrovascular function. These findings underscore the potential advantages of preserving or improving gait speed in promoting cognitive well-being and overall cardiovascular health [35, 36]. Furthermore, a lower incidence of movement disorders and reduced mortality rates has been closely linked to gait speed [37, 38].

A new observational study has found that walking is associated not only with aging in older adults, but also with accelerated apparent age and older faces in younger adulthood [39]. This suggests that walking may be a potential intervention for senescence rather than just an aging feature. The above observational studies have provided many new suggestions for the relationship between walking and aging, but they still have some observational limitations because they cannot provide clear causality due to the lack of powerful interventions. Our MR results can provide further valuable insight into the precise relationship between walking and the aging process in the current study, suggesting that walking acceleration may be a potential daily measure to slow the rate of aging.

MR studies stand out as a powerful approach for establishing causal relationships, particularly rooted in genetic factors. This potency is exemplified by the observed larger effects of associations between risk factors and health outcomes in MR studies compared to what is suggested by observational or interventional studies, as seen in contexts such as blood pressure and lipid levels [40–42]. This difference is attributed to MR studies measuring lifetime exposure, while observational studies capture exposure at a single time point, and interventional studies track changes over relatively short time frames [43].

The unique strengths of MR studies, including lifetime exposure measurement and extensive sample sizes, prove invaluable when investigating complex phenomena like gait speed. Routine intervention studies often struggle to achieve comparable sample sizes and comprehensive lifetime exposure data. Furthermore, the chronic effects of walking speed on the aging process pose challenges due to the lack of reliable markers based on age-related characteristics. MR studies, characterized by their genetic foundation and large-scale scope, bridge this research gap and provide a more thorough understanding of the causal relationship between walking speed and aging.

Horvath’s epigenetic clock, among the earliest methylation clocks, demonstrates a remarkable correlation between DNAm age and chronological age (0.96) [20, 44]. Renowned for its accuracy and versatility across diverse tissues and cell types, this model has been validated using hundreds of datasets [45]. Its applicability spans various tissues and organs, including whole blood, cerebellum, colon, kidney, liver, and lung. As a result, its acceleration serves as a reflective indicator of aging across different tissues within the body [20]. The Hannum epigenetic clock, based on 450 K methylation data from whole blood samples, complements Horvath’s epigenetic clock by exhibiting increased accuracy in predicting adult blood samples [19]. PhenoAge, constructed from 513 age-related CpG sites across three methylation chip platforms, distinguishes itself with cross-platform applicability, mortality risk differentiation among individuals of the same chronological age, and a stronger correlation with behavioral lifestyle compared to Horvath’s epigenetic clock [5]. GrimAge, featuring DNA methylation-based plasma protein markers and smoking pack-years, prioritizes lifestyle and age-related diseases, resulting in more accurate lifespan predictions [21]. Its acceleration mitigation by walking speed suggests an association between higher gait speed and lower mortality.

The investigation into the causal effects of walking on four classical epigenetic clocks provides a nuanced understanding of how walking influences accelerated aging. The emergence of the aging clock not only serves as a crucial assessment tool for aging research, but also presents an avenue for interventions aimed at delaying or potentially reversing the aging process. Our findings indicate that increasing daily walking speed may serve as a viable lifestyle strategy to slow down the aging process. However, the effects of walking duration and frequency on aging deceleration lack convincing evidence.

The study boasts technical and conceptual strengths, being the first to explore the bidirectional relationship between epigenetic aging acceleration and walking speed. Leveraging multiple large datasets, including summary statistics from the UK Biobank GWAS meta-analysis and apparent time-clock acceleration data from a substantial GWAS meta-analysis, enhances the robustness of our findings. However, limitations include the exclusive use of data from individuals of European ancestry, impacting population diversity, and the unknown mechanism through which walking affects aging, necessitating further exploration of potential mediators. Additionally, future studies are crucial to determining the specific relationship between different walking speeds and the degree of aging acceleration, paving the way for the development of more scientific anti-aging walking protocols.

## Conclusion

Our study demonstrates that a faster usual walking speed is significantly associated with a delay in epigenetic age acceleration. This finding highlights the potential benefits of maintaining a brisk walking pace for slowing down the aging process, as supported by our analysis of the four classical epigenetic ages.

### Supplementary Information

Supplementary file 1.

Supplementary file 2.

## Data Availability

The original contributions presented in the study are included in the article/Supplementary Material. Further inquiries can be directed to the corresponding authors.
